# Royal Commission on the Poor Laws

**Published:** 1905-12-02

**Authors:** 


### ROYAL COMMISSION ON THE POOR LAWS.

The King has been pleased to approve the appointment of a Royal Commission to inquire :—

1. Into the working of the laws relating to the relief of poor persons in the United Kingdom.

2. Into the various means which have been adopted outside of the Poor Laws for meeting distress arising from want of employment, particularly during periods of severe industrial depression; and to consider and report whether any, and if so what, modification of the Poor Laws or changes in their administration, or fresh legislation for dealing with distress are advisable.

The Commissioners will be :—

The Right Hon. Lord George Hamilton, M.P., Chairman.
The Right Hon. The O'Conor Don.
The Right Hon. Sir H. A. Robinson, K.C.B., Vice-President of the Local Government Board for Ireland.
The Right Hon. Charles Booth, F.R.S.
Sir Samuel Provis, K.C.B., Permanent Secretary to the Local Government Board for England.
Mr. F. H. Bentham.
Dr. A. Downes.
The Rev. T. Gage Gardiner.
Mr. George Lansbury.
Mr. C. S. Loch.
Mr. J. Patten Macdougall, Vice-President of the Local Government Board for Scotland.
Mr. T. Hancock Nunn.
The Rev. L. R. Phelps.
Professor William Smart.
The Rev. H. Russell Wakefield.
Mrs. Bernard Bosanquet.
Mrs. Sidney Webb.
Miss Octavia Hill.

Many of the members of the Royal Commission on the Poor Laws are already well known in connection with their parliamentary, departmental, or philanthropic work. Mr. Bentham has been for some time Chairman of the Bradford Board of Guardians, and he acted as President of the Poor Law Conference at the Guildhall. Dr. Downes is chief of the Poor Law Medical Department of the Local Government Board. The Rev. T. Gage Gardiner was one of the founders of Toynbee Hall, and was for some time rector of St. George's, Southwark, serving upon the Board of Guardians of St. Saviour's, and acting as Chairman of the local vestry. He afterwards became rector of Farnham, and has now resigned that living in order to devote his time to questions of Poor Law administration, on which he is an acknowledged authority. Some years ago he served as a Poor Law Guardian at Colchester. Mr. George Lansbury has come prominently before the public in connection with the unemployed question. He has been a Guardian of the Poor for several years, and has long been Chairman of the Poplar Board and of the House and School Committees. He was a member of Mr. Walter Long's Unemployed Committee for London. Mr. C. S. Loch is the Secretary of the Charity Organisation Society. Mr. T. Hancock Nunn has been connected with Toynbee Hall. He became a member of the Stepney Board of Guardians, and was associated with the Mansion House Fund of 1886. He has been connected with almost every Mansion House Unemployed Committee since that date, and was a member of the London County Council Committee on Want of Employment in 1903, and of Mr. Long's Committee. He now lives at Hampstead, where he is a Guardian and Borough Councillor. The Rev. L. R. Phelps is a Fellow and Tutor of Oriel College, Oxford, and is a well-known economist. Professor Smart, of Glasgow, is another well-known economist. The Rev. H. Russell Wakefield, rector of St. Mary, Bryanston Square, has been Mayor of Marylebone, and has served on Mr. Long's Committee. Mrs. Sidney Webb and Miss Octavia Hill have been prominently associated with Poor Law work. Mrs. Bosanquet is the wife of the Professor of Moral Philosophy at St. Andrews.

